# The role of HMGB1/RAGE/TLR4 signaling pathways in cigarette smoke‐induced inflammation in chronic obstructive pulmonary disease

**DOI:** 10.1002/iid3.711

**Published:** 2022-10-11

**Authors:** Ling Lin, Jing Li, Qing Song, Wei Cheng, Ping Chen

**Affiliations:** ^1^ Department of Respiratory and Critical Care Medicine, The Second Xiangya Hospital Central South University Changsha Hunan China; ^2^ Research Unit of Respiratory Disease Central South University Changsha Hunan China; ^3^ Diagnosis and Treatment Center of Respiratory Disease Central South University Changsha Hunan China

**Keywords:** chronic obstructive pulmonary disease, cigarette smoke, HMGB1, inflammation, RAGE, TLR4

## Abstract

Chronic obstructive pulmonary disease (COPD) is a common chronic respiratory disease with irreversible and continuous progression. It has become the fifth most burdensome disease and the third most deadly disease globally. Therefore, the prevention and treatment of COPD are urgent, and it is also important to clarify the pathogenesis of it. Smoking is the main and most common risk factor for COPD. Cigarette smoke (CS) can cause lung inflammation and other pathological mechanisms in the airways and lung tissue. Airway inflammation is one of the important mechanisms leading to the pathogenesis of COPD. Recent studies have shown that high mobility group box 1 (HMGB1) is involved in the occurrence and development of respiratory diseases, including COPD. HMGB1 is a typical damage‐associated molecular pattern (DAMP) protein, which mainly exerts its activity by binding to the receptor for advanced glycation end products (RAGE) and toll‐like receptor 4 (TLR4) and further participate in the process of airway inflammation. Studies have shown that the abnormal expression of HMGB1, RAGE, and TLR4 are related to inflammation in COPD. Herein, we discuss the roles of HMGB1, RAGE, and TLR4 in CS/cigarette smoke extract‐induced inflammation in COPD, providing a new target for the diagnosis, treatment and prevention of COPD.

## Graphical abstract

High mobility group box 1 (HMGB1) is a typical damage‐associated molecular pattern protein, which mainly exerts its activity by binding to the receptor for advanced glycation end products (RAGE) and toll‐like receptor 4 (TLR4) and further participates in the process of airway inflammation. We discussed the role of HMGB1, RAGE, and TLR4 in the cigarette smoke/Cigarette smoke extract‐induced inflammation of COPD, providing a new target for the diagnosis, treatment and prevention of COPD.

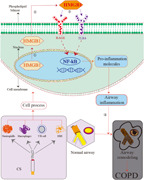

## 1 INTRODUCTION

Chronic obstructive pulmonary disease (COPD) is a chronic respiratory disease in which the airway structure is remodeled due to long‐term inflammation of the airway and lungs_._ ^1^, ^2^, ^3^ It usually manifests as persistent airflow limitation and respiratory symptoms.^4^ It will seriously affect the quality of life and physical health of COPD patients. Because of the high morbidity, disability and mortality of COPD, it has become a disease of global concern.^5^ Although there are treatments such as corticosteroids, long‐acting muscarinic antagonist, long‐actingβ‐2‐agonist, and so forth, available, they only provide short‐term benefits and do not stop COPD progression. In addition to these classic therapeutics, studies have shown some novel therapeutics that target inflammation in COPD, such as p38 MAPK inhibitors, cytokine inhibitors, chemokine receptor antagonists (PPARγagonizts), anti‐oxidants, and so forth.^6,7^ However, the exact pathogenesis and treatment of COPD are not very clear.

The most common causes of airflow obstruction include cigarette smoke (CS), occupational and environmental exposures and a history of tuberculosis.^8^ Cigarette smoke contains many harmful ingredients and has a damaging effect on the respiratory tract. Studies have shown that long‐term smoking can not only destroy the structure of the airway wall, but also increase the secretion of mucous glands and cause obstructive bronchitis.^9^ Therefore, it has become one of the important reasons for the development of COPD. Moreover, CS can stimulate the lung tissue to produce a large amount of reactive oxygen species (ROS), which activated the production and release of damage‐associated molecular patterns (DAMPs) and inflammatory factors, thereby causing lung inflammation.^10^, ^11^

Airway inflammation is one of the important mechanisms of COPD, and high mobility group box 1 (HMGB1) is considered to be one of the main immune inflammation factors involved in various tissues, including airways_._ ^12^, ^13^ HMGB1 is a typical alarm element and a typical DAMP molecule. It contains three domains, consisting of boxes A and B separated in series by a short and flexible linker and a C‐terminal tail containing 30 glutamate or aspartate residues. Box A and box B are the DNA binding domains of HMGB1; their negatively charged tails can bind to histones. HMGB1 utilizes a variety of membrane receptors in its signaling cascade. It can bind to the receptor for advanced glycation end products (RAGE) occurs and toll‐like receptor 4 (TLR4) at box B of the HMGB1 molecule. Importantly, HMGB1 is one of the important molecules that cause pathological changes in COPD, as its main role is to cause airway inflammation.^14^, ^15^, ^16^ HMGB1 can act as a chemotactic or proinflammatory mediator in the formation of COPD airway inflammation by directly combining RAGE and TLR4.^14^, ^17^, ^18^ At the same time, RAGE and TLR4 are also immune cell receptors, and thus can regulate the immune inflammatory response of the airway epithelium.^19^, ^20^, ^21^, ^22^

More and more evidence indicates that the HMGB1, RAGE, and TLR4 signaling pathways play a crucial role in COPD inflammation. Upregulation of HMGB1, RAGE, and TLR4 protein levels were detected in lung tissue of COPD smoker, bronchoalveolar lavage fluid (BALF), serum and other samples. However, inhibiting the expression of this pathway or its components can alleviate the degree of inflammation.^23^, ^24^, ^25^, ^26^ Herein, we discuss the role of HMGB1, RAGE, and TLR4 in the cigarette smoke‐induced inflammatory response in COPD.

## 2 PRESENT FORMS AND RELEASE OF HMGB1

### 2.1 Different forms of HMGB1

HMGB1 has several presence forms, including nuclear HMGB1, cytosolic HMGB1, membrane HMGB1, and extracellular HMGB1. Nuclear HMGB1 acts as a DNA chaperone with DNA binding and bending activities and regulates a number of critical DNA events. HMGB1 is not only involved in DNA replication,^27^, ^28^ but also plays a role in the early stages of DNA repair^29^ and gene delivery.^30^

HMGB1 has two nuclear localization sequences (NLS) located in box A (AA 28‐44) and between box B and the tail of the C‐terminal (AA 179−185), respectively.^31^ The acetylation of multiple lysine residues in the NLSs promotes HMGB1 translocation from the nucleus to the cytoplasm and subsequent release into the extracellular environment.^31^, ^32^

Currently, we know that HMGB1 is normally located in the nucleus and translocates from the nucleus to the cytosol under various stressors. The main function of HMGB1 in the cytoplasm is to act as a regulator of autophagy^33^ and exert functiona in inflammation.^34^

HMGB1 has been reported to be present on cell surface membranes involved in neurite outgrowth,^35^ platelet activation,^35^, ^36^ cell differentiation,^37^ innate immunity.^38^

HMGB1 can be secreted by immune cells or passively released by dead, dying, or injured cells to become extracellular HMGB1. It has multiple activities and is involved in several processes such as inflammation,^39^ immunity,^40^ migration,^41^ and cell death^42^ and so forth. The various forms of HMGB1 are all involved in the inflammatory pathological process of COPD.

### 2.2 Intracellular and extracellular interaction of HMGB1 in COPD

HMGB1 is a nuclear nonhistone binding protein that can shuttle between the nucleus and the cytosol through nuclear pores. NLS are posttranslationally modified by hyperacetylating lysine residues within NLS1 and NLS2.^31^ Under conditions of oxidative stress, Hyperacetylation of NLS by histone acetyltransferase (p300, p300/CBP‐associated factor [PCAF], CREB binding protein [CBP]) is required to induce nucleocytoplasmic translocation.^31^ HMGB1 also translocates from the nucleus to the cytoplasm through nuclear export factor 1 (CRM1).^43^ Cytoplasmic HMGB1 can then be secreted from the cell in secretory vesicle‐mediated exocytosis in injured or necrotic cells.^31^ Whether passively or actively secreted, HMGB1 then can accumulate in extracellular spaces such as the airways and circulating blood.^13^ Extracellular HMGB1 can bind to receptors on cell membranes in the airway and blood, that is, RAGE and TLR4, causing the release of intracellular proinflammatory factors, including TNF‐α, interleukin (IL)‐1β, and CXCL8, which leads to pulmonary airway inflammation and promotes the translocation of HMGB1 in the nucleus to the cytoplasm.^13^, ^44^ The cells secrete additional cytoplasmic HMGB1 from as extracellular HMGB1. When HMGB1 is oxidized, it forms a disulfide bond between C23 and C45, which imparts chemokine and cytokine activity.^45^

### 2.3 Mechanism of HMGB1 release

The release of HMGB1 from the nucleus is so important that it triggers downstream biological processes, such as autophagy, immune inflammation, cell differentiation, angiogenesis, and cell migration.

When exposed to infectious factors such as bacteria and viruses, endogenous signals including H_2_O_2_ and NO, and metabolic abnormalities such as lipopolysaccharide (LPS) and extracellular adenosine triphosphate, the immune cells, fibroblasts, endothelial cells, and epithelial cells of the lung actively release HMGB1.^46^ Active secretion of HMGB1 is also affected by posttranslational modifications (PTM). Bonaldi et al.^31^ first demonstrated that LPS stimulation of monocytes and macrophages leads to the acetylation of PCAF, CBP, and P300 sites in the two NLS of HMGB1, thereby activating cytoplasmic translocation and secretion of HMGB1 in monocytes. The interaction between CRM1 and HMGB1 was enhanced by various inflammatory stimuli, and the translocation of HMGB1 from nucleus to cytoplasm was significantly inhibited by CRM1 inhibitors.^43^ Besides, Besides, the reactive oxygen species (ROS) signaling pathway,^47^ the calcium signaling pathway^47^ and tumor necrosis factor (TNF)‐α dependent mechanisms^48^ are also involved in the active release of HMGB1.

Damaged or necrotic cells may secrete HMGB1 passively during cell death processes such as necrosis, apoptosis, cell death, and autophagy, or when cells are damaged by various stimuli. Many signaling pathways are involved in this process. Huang et al.^49^ demonstrated that PARP1 mediates the release of HMGB1 during necrosis. Deletion of Peroxisome proliferator‐activated receptors (PARP1) or use of PARP inhibitors significantly inhibited DNA damage‐induced HMGB1 translocation and release. Besides, other molecular, including receptor‐interacting serine‐threonine kinase (RIP3),^50^ caspase family^51^ and others are also involved in this process.

Therefore, after being stimulated by various infectious factors, HMGB1 will be actively or passively secreted from the nucleus, thus participating in the occurrence of inflammation in COPD.

## 3 THE ROLE OF HMGB1 IN CIGARETTE SMOKE‐INDUCED INFLAMMATION IN COPD

During the process of cell death, that is, necrosis or apoptosis, HMGB1 is released from the nucleus to the outside of the cell, and it binds to its corresponding receptors after posttranslational modification, mainly RAGE and TLR4, causing a series of reactions. HMGB1 is mainly involved in the formation of airway inflammation in COPD.

### 3.1 Evidence for HMGB1 involvement in cigarette smoke‐induced inflammation in COPD

Recently, studies conducted in patients and experimental models have shown that HMGB1 played an important role in the pathogenesis of COPD. HMGB1 was significantly upregulated in the blood and lung tissues of nine COPD smokers compared with eight smokers without COPD and nine healthy subjects. It was shown that HMGB1 positive cells were significantly observed in the epithelium, submucosal area and alveoli of COPD smokers. However, no significant positive expression was found in the lung tissue of the two latter groups.^52^ Higher expression of HMGB1 was also found in the induced sputum of COPD patients.^53^ In addition, the level of HMGB1 in the BALF and epithelial lining fluid was also associated with lung function in patients with COPD.^25^, ^54^ The higher of the HMGB1level, the worse of the lung function was, including the lower FEV1 and FEV1/FVC in patients with premedication and after medication. Besides, the emphysema index in HRCT was also correlated with HMGB1 levels in the peripheral airways. Cytokines play a major role in airway inflammation in COPD patients, and HMGB1 in BALF was positively correlated with airway IL‐1β.^25^, ^54^ In the airway, more active ROS response, as well as the platelet activating factor receptor (PAFR) level was increased in COPD patients compared with nonsmoker healthy controls. PAFR further intensified the recruitment of inflammatory cells, the production of pro‐inflammatory cytokines and angiogenesis. However, HMGB1 levels were significantly increased in cigarette smoke extract (CSE)‐induced neutrophils with high expression of PAFR. Therefore, in the process of CS promoting the ROS response, the activation of PAFR also increased the expression of HMGB1.^55^

Some studies in animal models have demonstrated that abnormally expressed HMGB1 levels were related to the onset of CS‐related COPD. Mice exposed to cigarette smoke develop an inflammatory response in the respiratory tract that leads to emphysema. After mice were exposed to 12 cigarettes/day for 60 days and control mice were exposed to normal air, alveolar macrophages and neutrophils increased in the BALF of CS‐exposed animals, and the expression of matrix metalloproteinases (MMP)−12 a HMGB1 in lung tissue was also upregulated, while the activity of antioxidant enzymes was significantly decreased compared to the control group.^56^ Cheng et al.^44^ found upregulated TLR4 expression in the lung tissues of mice exposed to cigarette smoke and upregulated HMGB1 levels in lung tissues and airways due to extracellular translocation from the cytoplasm. However, inhibiting the expression of HMGB1 was found to reduce airway inflammation in mice. Exogenous HMGB1 increased proinflammatory cytokine production in wild‐type mice, but it did not affect proinflammatory cytokine production in TLR4‐KO mice.^44^ These results suggested that TLR4 signaling is responsible for HMGB1‐induced proinflammatory cytokine production in lung tissue. Besides, HMGB1 can be directly regulated by the nuclear factor‐κB (NF‐κB). After treatment with CS and LPS, the NF‐κB inhibitor, pyrrolidine dithiocarbamate (PDTC) could decrease inflammatory cell infiltration and improve emphysema and lung bullae wall fracture in rats.^57^ In addition, in the LPS‐induced and fungus‐induced rat emphysema model, the expression of HMGB1 was significantly increased in the lung tissue and alveolar macrophages derived from the lung tissue of the emphysema mice, as were the levels of inflammatory mediators including TNF‐α, IL‐1β, IL‐6, and IL‐33. Compared with alveolar macrophages from control mice, CS and *Aspergillus fumigatus* resulted in increased levels of myeloiddifferentiationfactor88 (MyD88), p‐p65, p‐Spleentyrosinekinase (Syk), Phosphatidylinositide 3‐kinases (PI3K), and NF‐κB in alveolar macrophages. However, this response was decreased after HMGB1 siRNA or PDTC treatment. The expression levels of TNF‐α, IL‐1β, and IL‐6 also were downregulated.^18^ These data indicate that the function of HMGB1 in the COPD alveolar macrophage immune response may be achieved by activating MyD88/NF‐κB and Syk/PI3K signals.

In several vitro experiments, it was also shown that HMGB1 was associated with the pathogenesis of COPD. Heijink et al.^58^ showed that CS exposure can induce neutrophil necrosis and lead to the release of and HMGB1 other related DAMPs, which amplifies CS‐induced airway inflammation by promoting an airway epithelial proinflammatory response. Macrophages exposed to CSE stimulate the expression of HMGB1 and release it to the extracellular space.^59^ In addition, CS‐induced translocation and release of HMGB1 induced migration and NF‐κB activation by inducing autophagy in lung macrophages.^60^ After PM2.5 treatment, the secretion of HMGB1 and the expression of RAGE in epithelial cells increased, accompanied by the activation and migration of NF‐κB into the nucleus and higher expression of airway fibrosis‐related factors, such as Platelet‐Derived Growth Factors (PDGF)‐AB, PDGF‐BB, and TGF‐β1. Inhibition of HMGB1 expression or treatment with a RAGE antibody was found to inhibit these processes, indicating that HMGB1 may contribute to airway inflammation and then cause airway remodeling in COPD.^61^ After A549 cells were transfected with an HMGB1 plasmid, TNF‐α and macrophage inflammatory protein (MIP)−2 messenger RNA (mRNA) levels were upregulated, and matrix metalloproteinase (MMP)−7 protein was also increased in the supernatant.^62^ The expression of miRNA‐30c increased at the same time, accompanied by abnormal cell damage and apoptosis. This result indicates that HMGB1 has the potential to induce alveolar apoptosis in addition to airway inflammation in COPD.^62^ After TLR4‐KO mice were exposed to cigarette smoke or mice were treated with a HMGB1 polyclonal antibody, the levels of inflammatory factors in the airways were significantly reduced. Further in vitro experiments performed to study downstream inflammation pathway signals found that airway HMGB1 can activate the NF‐κB and JNK/p38 pathways through TLR4 and MyD88‐mediated pathways, resulting in airway inflammation^44^ (Table 1).

**Table 1:** Implication of HMGB1 in CS‐induced COPD inflammation

	E group	C group	Downstream signal	Observations	References
1	30 smokers with COPD	20 never‐smokers	RAGE	E group showing higher HMGB1, BALFF HMGB1 correlated positively with IL‐1βand negatively with FEV(1)	Ferhani et al.^25^
				E group showing RAGE overexpressed in the airway epithelium and smooth muscle and colocalized with HMGB1	
2	11 smokers with COPD	Eight Smokers without COPD; 9 nonsmokers healthy controls		E group showing HMGB1‐expressing cells in epithelium and submucosal areas increased	Ko et al.^52^
				Plasma HMGB1 levels negatively correlated with post‐bronchodilator FEV1/FVC ratio (*r* = −0.585) in smokers	
3	C57BL/6 J mice + CS	C57BL/6 J mice + AIR	TLR4/MyD88 to NF‐κB and JNK/p38 pathways	E group showing inflammatory response increased	Cheng et al.^44^
				Expression of HMGB1, TLR4, MyD88, and proinflammatory cytokines (TNF‐α, IL‐1β, IL‐6, IL‐8, IFN‐γ, MCP‐1）increased	
4	BALB/C mice + CS + macrophages stimulated with *Aspergillus fumigatus* conidia	BALB/C mice + AIR	Dectin‐1, TLR2/4MyD88/NF‐κB and syk/PI3K	E group showing inflammatory response increased	Zhang et al.^18^
				HMGB1, Dectin‐1, TLR2/4, MyD88, PI3K, syk increased	
5	Rats + CS + LPS	Rats + AIR + PBS	NF‐κB	E group showing inflammatory cell infiltration increased and lung bullae wall fracture was formed	Wang et al.^57^
				HMGB1 and NF‐κB increased	
6	Rats + CS	Rats + AIR	NF‐κB	E group showing airway inflammation and	Xu et al.^63^
				Caspase‐3, bax and c‐Jun, HMGB1 increased and reversed by NF‐κB inhibitors	
7	Rats + CS+ LPS	Rats + AIR + PBS	TLR4, MyD88, TRAF‐6, LOX‐1	E group showing FEV and FVC decaeased	Liu et al.^64^
				mRNA and protein of TLR4, MyD88, TRAF‐6, LOX‐1, and HMGB1 increased	
				There is a significant negative correlation between FEV, and TLR4 and HMGB1 expression levels	
8	A549 cells + rHMGB1	A549 cells		E group showing cell injury and apoptosis increased	Nagayasu et al.^62^
				HMGB1, TNF‐α, MIP‐2, MMP7, CCNA2, and PTEN proteins overexpressed	

Abbreviations: AIR, fresh air; C group, control group; COPD, chronic obstructive pulmonary disease; CS, cigarette smoke; CSE, cigarette smoke extract; E group, experimental group; HMGB1, high mobility group box 1; LPS, lipopolysaccharide; MAPK, mitogen‐activated protein kinase; NF‐κB, nuclear factor‐κB; PBS, phosphate buffer saline; RAGE, the receptor for advanced glycation end products.

### 3.2 Potential for HMGB1 to be a clinical biomarker associated with inflammation in COPD

Serum HMGB1 and RAGE levels are significantly higher when COPD patients experienced acute exacerbations compared with stable period. It is worth noting that HMGB1 levels are significantly higher in women, and there is a significant difference between COPD patients with and without airway infection during the exacerbation phase. The increase in serum HMGB1 levels is less in patients with airway infection.^65^ This is an interesting phenomenon that deserves further investigation. Plasma HMGB1 and the soluble form of RAGE (sRAGE) also decrease upon recovery from an acute exacerbation.^66^ This suggests that the level of HMGB1 in the airway may be a marker of COPD severity and progression. HMGB1 could become a clinical biomarker for the occurrence and development of COPD.

## 4 THE ROLE OF RAGE IN CIGARETTE SMOKE‐INDUCED INFLAMMATION IN COPD

RAGE belongs to the immunoglobulin superfamily and has various forms and is widely expressed in a variety of cell types and tissues, including vascular cells (endothelial cells and smooth muscle cells) and immune/inflammatory cells (neutrophils, monocytes/macrophages, lymphocytes, and dendritic cells). Interestingly, the expression of RAGE increases during ageing, especially in the lungs.^67^ RAGE binds to several DAMPs (including AGEs, HMGB1, S100, and LPS) to mediate different cellular responses, especially the innate immune response. The multi‐ligand receptor RAGE has the unique property of recognizing the three‐dimensional structure of a ligand rather than a specific amino acids, and is therefore known as a pattern recognition receptor.^68^

### 4.1 Evidence for RAGE implication in cigarette smoke‐induced inflammation in COPD

Ferhani et al.^25^ found that RAGE was overexpressed in airway epithelium and smooth muscle of COPD patients and co‐localized with HMGB1. In addition, RAGE and its common ligands (HMGB1, S100A8, S100A9, and S100A12) were activated in human bronchial epithelial cells after CS stimulation; RAGE binds to these ligands to induce immune responses. Moreover, the expression of RAGE was also positively correlated with CS‐induced airway inflammation.^69^, ^70^ It was shown that RAGE is also one of the important molecules involved in the formation of COPD airway inflammation.

RAGE is involved in the pathological process of COPD in animal models as well. RAGE and its ligand S100A were upregulated in wild type mice exposed to CS, and mediated airway inflammation and oxidative stress injury. Stogsdill et al.^71^ found that, compared with control mice, dilation of alveolar spaces, and increased apoptotic cells were observed in RAGE transgenic mice, along with higher vascular permeability and the number of inflammatory cells in the BALF and increased expression of MIP‐2 and IFN‐γ in lung tissue. This led to the degradation of lung elastase and an increase in MMP‐9, which promoted the process of airway remodeling. However, the administration of RAGE inhibitors to mice alleviated RAGE‐DAMP signaling suppressed the development and progression of emphysema.^72^ RAGE knockout mice require a longer period of time to develop emphysema due to reduced accumulation of neutrophils, as well as lower levels of the oxidative stress‐related protein thioredoxin reductase 1, heat shock protein 1, and other inflammatory factors. It therefore reduced airway inflammation and remodeling.^23^, ^73^

In CSE‐induced cells, both the expression of RAGE and RAS, a small GTPase that perpetuates proinflammatory signaling increase. In addition, CSE‐treated cells activate Nuclear factor erythroid2‐related factor 2 (Nrf2) to change the distribution and expression of RAGE, and then through MAPK and NF‐κB to induce redox‐sensitive DAMPs^74^ (Table 2), thereby participating in COPD complications. When the expression of RAGE in alveolar macrophages was downregulated, the important conduction intermediates p38, MAPK, and NF‐κB were downregulated, and the expression of related inflammatory factors TNF‐α and IL‐1β was also reduced.^75^

**Table 2:** Implication of RAGE in CS‐induced COPD inflammation

	E group	C group	Downstream signal	Observations	References
1	AKR mice + CS	AKR mice + AIR	Nrf2, NF‐κB	E group showing inflammatory response and Oxidative Stress and ER stress increased	Sanders et al.^23^
				Expression of HMGB1, RAGE, Nrf2, NF‐κB increased	
2	C57BL/6 + CS	C57BL/6 + AIR	DAM (RelA/p65 NF‐κB, JNK1/2, ERK1/2, and p38 MAPK) and Nrf2	E group showing inflammatory response and oxidative stress increased	Lee et al.^74^
				RAGE, DAMP, and Nrf2 increased	
3	Alveolar macrophages + CSE	Alveolar macrophages + PBS	p38, MAPK, and NF‐κB	E group showing inflammatory response increased	Robinson et al.^75^
				RAGE, p38, MAPK, and NF‐κB increased	
4	R3/1 cells + CSE	R3/1 cells + PBS	Ras/NF‐κB	E group showing inflammatory response increased	Reynolds et al.^76^
				RAGE, Ras, NF‐κB increased	
5	BEAS‐2B + CSE	BEAS‐2B + PBS	Nrf2	E group showing inflammatory response increased and Cellular Antioxidant Defense System was injured	Lee et al.^72^
				RAGE, Nrf2 increased	
				These were allievated by RAGE inhibitor (FPS‐ZM)	

Abbreviations: AIR, fresh air; C group, control group; COPD, chronic obstructive pulmonary disease; CS, cigarette smoke; CSE, cigarette smoke extract; E group, experimental group; HMGB1, high mobility group box 1; LPS, lipopolysaccharide; MAPK, mitogen‐activated protein kinase; NF‐κB, nuclear factor‐κB; PBS, phosphate buffer saline; RAGE, the receptor for advanced glycation end products.

Genetic alterlations and PTM of RAGE were also involved in the development of COPD. As previously explored, the AGER gene, located at 6p.21.3, encodes for RAGE. Oxidative and inflammatory stress are known inducers of the highly polymorphic AGER gene,^77^ and thus participated in the pathogenesis of COPD.^78^, ^79^ Single nucleotide polymorphisms (SNPs) of the AGER gene have been correlated with the release of sRAGE,^80^ resulting in the formation of airway inflammation. The related and common mutation sites are RS2071288, RS2070600, RS1800625, RS1800624, and rs184003.^77^, ^81^, ^82^, ^83^ In addition, RAGE mediates the PTM of functional genes, and is also involved in COPD onset. Li et al.^84^ found that the chemokine CXCL1, TLR6, and oncostatin M were hypomethylated in the promoter regions with less severe airway inflammation caused by CS exposure after the RAGE gene was knocked out. The specific mechanism is still unclear and needs further study (Table 2).

### 4.2 Potential for RAGE to be a clinical biomarker associated with inflammation in COPD

sRAGE acts as a natural antagonist of RAGE signaling because it isolates the RAGE ligand and inhibits RAGE‐dependent cellular responses.^85^, ^86^ The levels of sRAGE in serum, BALF and lung tissue are reduced in COPD patients with a smoking history.^87^, ^88^ Miniati et al.^88^ found that sRAGE was negatively correlated with the severity of emphysema. The lower the systemic level of sRAGE, the more severe the airflow limitation, including a lower FEV1% and a greater degree of emphysema on CT. Iwamoto et al.^89^ analyzed the association of plasma sRAGE and HMGB1 levels with longitudinal declines in lung function during a 4‐year follow‐up study. Not only was the baseline plasma sRAGE concentration an independent predictor of declines in FEV1/FVC in all groups, but reduced sRAGE levels were also significantly associated with faster declines in FEV1/FVC in smokers with COPD.^86^ These results suggest that sRAGE is a potential marker of COPD progression.

## 5 THE ROLE OF TLR4 IN CIGARETTE SMOKE INDUCED‐INFLAMMATION IN COPD

TLRs are innate immune receptors that detect the presence of conserved molecular patterns in pathogens, known as self‐derived molecules released in response to tissue damage (DAMPs). There are 10 types in humans and 12 in mice.^90^ TLR4 is a type I membrane glycoprotein and was the first one discovered in humans.^90^ It has been widely explored in the context of COPD pathology.^91^ TLR4 is expressed by congenital cells (neutrophils, macrophages, dendritic cells, endothelial cells, epithelial cells of the skin and mucous membranes) and adaptive immune cells. It has been widely explored in the context of COPD.^91^

### 5.1 Evidence for TLR4 involved in cigarette smoke‐induced inflammation in COPD

Pace et al.^92^ found that, compared with nonsmokers and non‐COPD patients, the number of neutrophils and chemotactic activity of BALF in COPD patients was increased, as was the TLR4 level,^92^ along with increased expression of TLR4 in the bronchial epithelium of COPD patients. More importantly, the overexpression of TLR4 and NOD1 in the bronchial epithelium in severe/very severe stable COPD was not only positively associated with CD4^+^/CD8^+^ cells and airflow obstruction, but also with bronchial inflammation and increased *Pseudomonas aeruginosa* bacterial load.^93^ This showed that TLR4 is involved in the process of airway inflammation caused by infection.

There are also some animal models and in vitro experimental results that illustrate how TLR4 is involved in the pathological process of airway inflammation and oxidative stress in smoking‐related COPD. In an in vivo study, CS exposure increased the levels of monocyte chemokine protein‐1 (MCP‐1), TNF‐α in BALF and TLR4/TLR2 in the lung of wildtype mice, accompanied by considerable infiltration of macrophages, neutrophils, lymphocytes, and dendritic cells into the lungs. However, the expression levels of inflammatory factors in the BALF were lower in TLR4 knockdown mice than in control mice after CS exposure.^94^ Doz et al.^95^ found that heat shock protein 70, a known TLR4 agonist, is induced in the airways during exposure to smoke, which may activate the innate immune system through TLR4/MyD88 and lead to airway inflammation. The added recruitment of neutrophils into the bronchoalveolar space and lung parenchyma of mice exposed to CS was decreased in TLR4, MyD88, and IL‐1R1 deficient mice.

The expression of TLR4 and p21 was increased in 16‐HBE cells stimulated with CSE, and IL‐8 mRNA neutrophil migration was upregulated. This was alleviated by carbocysteine.^96^ The expression of TLR4 was increased in human bronchial epithelial cell treated with CSE and LPS and further induced inflammation through upregulation of extracellular regulated protein kinases (ERK)1/2 and IkBa phosphorylation.^97^ MiR‐27‐3p regulates TLR2/4 intracellular signal transduction in alveolar macrophages to regulate the production of pro‐inflammatory cytokines and the polarization of alveolar macrophages.^98^

T cells also express TLR4, and CS exposure causes the activation of CD8^+^ T cells to produce IL‐1β, IL‐10, p70, TNF‐α, and other inflammatory factors; inhibiting the expression of TLR4 or TLR9 in CD8^+^ T cells significantly reduced the production of TNF‐α and IL‐10.^99^ Knockdown of Circ‐Hace1 expression in human bronchial epithelial cells treated with CSE reduced cell viability. Apoptosis, the inflammatory response and oxidative stress were also achieved by targeting TLR4, the downstream target of Mir‐485‐3p^100^ (Table 3). SNPs of TLR4 are related to COPD inflammation in COPD patients, and are related to worse lung function in COPD patients and an increase in the number of inflammatory cells in the sputum. These SNPs are involved in the local inflammatory response in the initial stage of the disease and the further development of the disease.^102^, ^104^ Polymorphisms are also associated with the risk of COPD development and exacerbation.^104^, ^105^ The mutation site RS11536889 in the 3'‐UTR of the TLR4 mRNA increases the expression levels of IL‐8, IL‐6 and TNF‐α on the protein level in the lung.^106^

**Table 3:** Implication of TLR4 in CS‐induced COPD inflammation

	E group	C Group	Downstream signal	Observations	References
1	10 smokers with COPD	10 nonsmokers without COPD	MyD88/IRAK and TRIF/IKKε/TBK1	E group showing systemic defects in TLR pathways in CD4^+^/Th1 cells	Knobloch et al.^101^
				MyD88/IRAK signal switch to TRIF/IKKε/TBK1 pathway	
2	Rats + CS + LPS	Rats + AIR + PBS	MyD88, TRAF‐6, LOX‐1	E group showing FEV and FVC decreased	Liu et al.^64^
				mRNA and protein of TLR4, MyD88, TRAF‐6, LOX‐1, and HMGB1 increased	
3	C57BL/6 + CS	C57BL/6 + AIR	MyD88, IL‐1R1	E group showing inflammatory response and oxidative stress increased	Doz et al.^95^
				TLR4/MyD88/IL‐1R1 upreguated	
4	Rats + CS	Rats + AIR	MyD88‐JNK/p3, NF‐κB	E group showing lung inflammation increased and bad pulmonary ventilation function	Cheng et al.^44^
				E group showing the abnormal activation of the TLR4‐MyD88‐JNK/p38 pathway and NF‐κB increased	
5	HBE + CSE	HBE + PBS		E group showing more neutrophil chemotactic migration, allievated by Carbocysteine	Pace et al.^96^
6	HBE + CSE + LPS	HBE + PBS	ERK1/2, IkBa	Cilomilast reduced TLR4 expression, IL‐8 release, and E group shwiing neutrophil chemotactic activity	Pace et al.^97^
				TLR4, ERK1/2, and IkBa phosphorylation increased	

Abbreviations: AIR, fresh air; C group, control group; COPD, chronic obstructive pulmonary disease; CS, cigarette smoke; CSE, cigarette smoke extract; E group, experimental group; HMGB1, high mobility group box 1; LPS, lipopolysaccharide; MAPK, mitogen‐activated protein kinase; NF‐κB, nuclear factor‐κB; PBS, phosphate buffer saline; TLR4: toll‐like receptor.

### 5.2 Potential for TLR4 to be a clinical biomarker associated with inflammation in COPD

Human peripheral blood mononuclear cells obtained from smokers have a higher TLR4 mRNA level than nonsmokers, but this decreases after 2 months of smoking abstinence in COPD patients.^107^ This suggests that quitting smoking can reduce TLR4 expression. In addition, the smoking index is related to TLR4 IL‐8 and MMP‐9 levels. This may help predict the future risk of developing this disease in smokers without using TLR4.^107^ Di Stefano et al.^93^ detected the expression of some members of the TLR family and inflammatory factors in the airway secretions of patients with severe COPD in a stable stage, accompanied by airway inflammation and bacterial infection.

## 6 THE PIVOTAL ROLE OF HMGB1 AND RAGE/TLR4 SIGNALING IN CIGARETTE SMOKE INDUCED INFLAMMATION IN COPD

**Figure 1:**
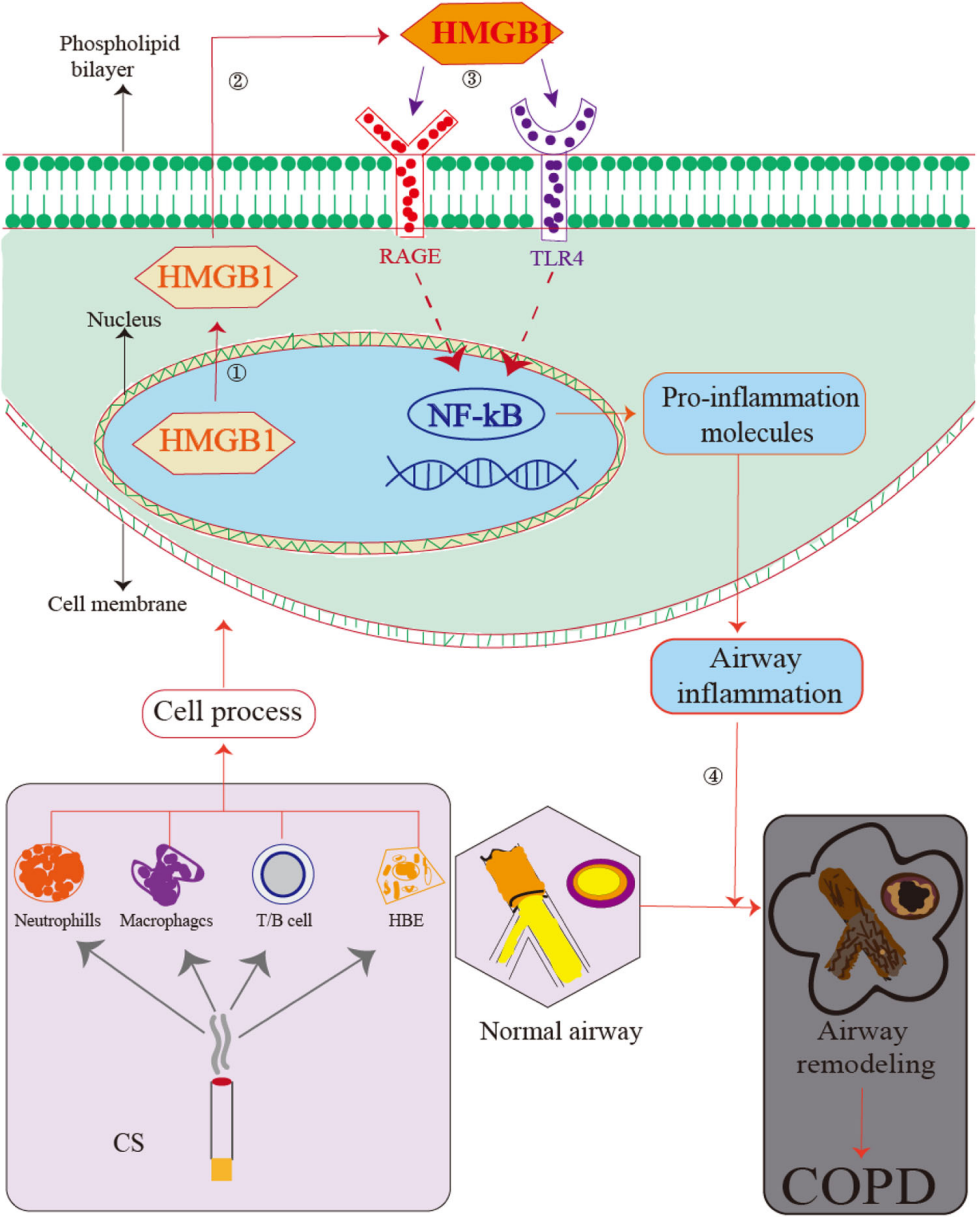
HMGB1 binding to RAGE and/or TLR4 to intiate airway inflammation and involve the development of COPD. When the airway is affected by cigarettes or other irritants, neutrophils, alveolar macrophages, lymphocytes and epithelial cells in the airway secreted HMGB1 actively. HMGB1 is transferred from the nucleus to the cytoplasm (1). This process often undergoes posttranslational Modifications, such as acetylation or phosphorylation. HMGB1 is secreted out of the cell through exocytosis (2). Outside the cell, HMGB1 can activate and bind its receptors RAGE and TLR4, leading to nuclear activation and translocation of NF‐κB(3). It leads to the production and release of inflammatory factors and chemokines, initiating airway inflammation. Sustained inflammation stimulus changes the airway structure and participates in the process of COPD (4). COPD, chronic obstructive pulmonary disease; HMGB1, high mobility group box 1; RAGE, the receptor for advanced glycation end products; TLR4: toll‐like receptor.

In the process of COPD inflammation, HMGB1 binds to its receptors RAGE and TLR4 to trigger the activation of a series of downstream signals, which triggers the process of airway inflammation and oxidative stress. The continuous accumulation of inflammation leads to changes in the structure of the airway. As a result, airway remodeling leads to the characteristics of airflow limitation in COPD (Figure 1).

After being stimulated by cigarettes, inflammatory cells such as alveolar macrophages, neutrophils, CD4^+^/CD8^+^ T cells and alveolar epithelial cells are damaged, causing HMGB1 to be released from the nucleus to the cytoplasm or even outside the cell. Therefore, the expression of intracellular HMGB1 increases. Intracellular signals induced by HMGB1 activate immune responses through binding with RAGE and/or TLR4, such as subsequent NF‐kB activation and translocation^108^ and MAPK and c‐Jun N‐terminal protein kainse (JNK) pathways‐mediated inflammation.^74^

Smoking can up‐regulate the expression of growth response gene 1 (EGR‐1) in the lung, and also stimulate the activation of the RAGE promoter and promote the RAGE expression.^109^ HMGB1 induces the activation of NF‐κB by binding to its receptor RAGE to trigger the production and release of downstream inflammatory factors IL‐1β, TNF‐α, and IL‐6. It can also activate the signal transducer, MAPK, RAS, AKT, and NF‐κB to initiate the inflammatory response process after HMGB1 binding with RAGE.^73^, ^75^, ^76^, ^110^ Besides, it can induce release of Nrf2, Egr1, and CCL2.^26^, ^78^ These processes result in the upregulated expression of TNF, IL‐1β, IFN‐γ, and related DAMPs.^23^, ^74^ This reaction raises the levels of downstream NO, MIP‐2, and endogenous nitrite, increases superoxide dismutase (SOD) activity and GSH/oxidized glutathione, causing an increase in oxidative stress and promoting inflammation.^73^ RAGE can also directly affect the hypomethylation of inflammatory chemokines such as CXCL1 to participate in the inflammatory process of the airway.^84^

In addition, HMGB1 can also bind to its receptor TLR4 to directly initiate the activation of downstream inflammatory factors such as IL‐Iβ and TNF‐α. In addition, it induces increased the expression of ERK1/2 and IBα phosphorylation after HMGB1 binds with TLR4.^97^ Besides, TLR4 activation can initiate the MyD88/IRAK and TRIF/IKKε/TBK1 pathways dependent signaling pathways.^101^ MyD88‐dependent signaling cascade activates the NF‐κB and MAPK signaling pathway.^95^ The activation of peroxisome proliferator‐activated receptor γ occurs due to HMGB1 binding with TLR4.^98^ It further affects the production and release of airway inflammatory factors such as IL‐1β, TNF‐α, IL‐6, IFN‐, IL‐8, and MCP‐1 to participate in the process of airway inflammation.^18^, ^44^ Continuous injury and stimulation can also cause the accumulation of inflammatory factors such as MIP‐2 and IFN‐γ, neutrophils and other inflammatory cells. This results in apoptosis or even necrosis, and can further aggravate ROS and the secretion of elastase and MMP‐9.^55^, ^58^ As a result, it these events lead to changes in the airway structure, known as airway remodeling.^55^, ^111^ In addition, increased vascular permeability and inflammatory mediators induced by the HMGB1 pathway contribute to upregulated levels of the tight junction molecules angulin‐1, lipolysis stimulated lipoprotein receptor, three‐cell protein, and two‐cell tight junction molecule occludin. These act on TGF‐β1 and p63 and lead to airway remodeling.^74^, ^112^

In short, the changes to downstream inflammatory factors triggered by HMGB1 binding to RAGE or TLR4 play a key role in the process of CS‐induced COPD inflammation.

## 7 INHIBITION OR BLOCKADE OF HMGB1, RAGE, AND TLR4 AS A POTENTIAL THERAPY AGAINST COPD

Based on the evidence that HMGB1, RAGE and TLR4 are involved in inflammation in COPD, targeting their effect may help clarify the potential mechanisms related to inflammation in COPD. Recently, several HMGB1, RAGE and TLR4 blocking/inhibiting strategies have been shown to be effective in relieving inflammation in COPD in experimental studies, which will be discussed below.

### 7.1 Effects of HMGB1 inhibition against COPD inflammation

The available therapeutic strategies for blocking/inhibiting HMGB1 include anti‐HMGB1 antibodies, HMGB1 release inhibitor agents and specific HMGB1 inhibitor compounds.

The degree of lung inflammation in CS‐exposed mice treated with anti‐HMGB1 antibodies was improved compared with CS‐exposed wild mice.^60^ Inhibition of HMGB1 expression can inhibit the process of disease occurrence. Nontransfected siHMGB1 mice and COPD mice transfected with siHMGB1 were transfected with macrophages containing *A. fumigatus conidia* and exposed to CS. The latter mice had reduced TNF‐α, IL‐1β, and IL‐6 levels, which also reduced the airway immune response.^18^ Therefore, antibodies and targeting HMGB1 gene expression could be a potential treatment of inflammation in COPD.

In addition, inhibiting the release of HMGB1 from the nucleus and alleviating the expression of HMGB1 in lung tissues could play a certain therapeutic role. Inhibitors of NF‐κB not only affect the post‐translation status of HMGB1, but also inhibit the release of HMGB1 from the nucleus into the cytoplasm. In an animal model treated with LPS + CS, HMGB1 mRNA and protein were downregulated after treatment with NF‐κB inhibitors. In addition, inflammatory cell, infiltration of tracheal tube wall, smooth muscle hyperplasia, local muscle layer and pulmonary bullae wall rupture were alleviated.^57^

There are other specific compounds inhibiting HMGB1. Glycyrrhizin (GL) is a natural inhibitor of HMGB1 that directly binds to HMGB1 protein and inhibits its biological activity. GL decreased HMGB1 and RAGE in BALF, as well as proinflammatory factors, such as TNF‐α, IL‐1β, and IL‐6 levels. It also inhibited HMGB/TLR4 related downstream transcription factors in lung tissues and cells, such as NF‐kB, JNK, and ERK1/2, thereby alleviating lung inflammation.^113^ Besides, ulinastatin,^114^ ginsenoside Rb3,^115^ green tea extract,^116^ and other compounds can reduce inflammation in CS/CSE‐related COPD models by reducing the release and expression of HMGB1.

In other studies, bone marrow mesenchymal stem cell (BMSC) derived exosomes were found to reverse the expression of HMGB1 and NF‐κB. However, overexpression of HMGB1 abolished the effects that BMSC‐derived exosomes induced on downregulation of inflammatory factors and NF‐κB^63^ (Table 1). Endothelial microparticles derived from endothelial cells can reduce the expression of HMGB1 in epithelial cells. This downregulated the release of TNF‐α and IL‐1β and then attenuated CSE‐induced inflammation in HBE cells.^117^ The expression level of miR‐181a‐5p was lower in the lung tissues of COPD model mice. However, miR‐181a‐5p overexpression targets HMGB1 to inhibit the NF‐κB pathway, thus alleviating the inflammatory response in COPD mice.^118^

It is expected that these agents targeting HMGB1 will have a protective effect on lung inflammation, and thus may be a possible drug class for the treatment of COPD inflammation.

### 7.2 Effects of RAGE blockade in relieving COPD inflammation

Anti‐RAGE antibodies, specific inhibitors of RAGE and some compounds that can interfere with or inhibit RAGE expression and related signaling pathways are available. Therefore, RAGE may become a target for the treatment of COPD inflammation.

Blondonnet et al.^119^ found that anti‐RAGE antibody could improve lung injury and arterial oxygenation and reduce alveolar inflammation in mice exposed to CS. Blocking RAGE reduced the damage to lung tissue by reducing damage to alveolar type 1 epithelial cells.

The broadest and strongest evidence currently available to reduce RAGE‐mediated CS‐related inflammation is FPS‐ZM1, a RAGE inhibitor. FPS‐ZM1 treatment significantly reversed emphysema and reduced lung inflammation in Nrf2^+/+^ mice.^72^ Furthermore, after treating lung epithelial cells induced by CSE with FPS‐ZM1, the levels of endogenous nitrite, SOD activity, and the levels of glutathione (GSH)/oxidized glutathione and inflammatory factors were reduced. It specifically specifically inhibited the activation of redox sensitive DAMP signals in CSE‐induced human alveolar type II epithelial cells by Nrf2.^74^ Besides, tanshinone IIA^120^ and TTP488,^121^ orally bioavailable small molecule inhibitors of RAGE, could also alleviate inflammation and neuroinflammation. This may lead to further study into the reduction of RAGE‐related inflammation in COPD.

In addition, the use of MSC‐derived exosomes can also inhibit the release of DAMPs caused by CS exposure, such as S100A4 and S100A8, HMGB1, RAGE and AGE, and thus reduce lung inflammation in animal models of emphysema. In addition, the co‐culture of MSC and lung epithelial cells was found to reduce mitochondrial stress.^122^

Although the preclinical study of RAGE against COPD reinforces the fact that RAGE may be a potential therapeutic target, future preclinical and clinical studies are necessary to evaluate the safety and efficacy of RAGE antagonists in COPD inflammation.

### 7.3 Attenuateing the expression of TLR4 to relieve COPD inflammation

TLR4 activation can actively promote inflammation in the pathogenesis of COPD, therefore suggesting that decreasing the expression of TLR4 may alleviate inflammation in COPD.

Sulforaphane can inhibit the TLR4/MyD88 pathway and reduce the release of downstream inflammatory cytokines, suggesting that sulforaphane may have an anti‐inflammatory effect in COPD.^123^ By inhibiting the abnormal activation of the TLR4/MyD88 pathway, cyclic peptide extracts can reduce lung inflammation, increase alveolar space, improve inflammatory cell infiltration, and improve pulmonary ventilation function in COPD model rats.^64^ Moreover, cilomilast can reduce TLR4 expression and neutrophil chemotactic activity in human bronchial epithelial cells.^96^ Carbocysteine alleviates the expression of TLR4 and p21 in 16‐HBE cells stimulated by CSE and neutrophils.^95^

The expression of miR‐149‐3p was lower in smokers with COPD. CSE stimulation downregulated the expression of miR‐149‐3p and upregulated TLR‐4 and NF‐κB levels in THP‐1 cells. Overexpression of miR‐149‐3p inhibited the TLR‐4/NF‐κB signaling pathway and reduces the secretion of IL‐1β and TNF‐α.^124^

## 8 CONCLUSION

CS is one of the main causes of COPD; it induces lung inflammation to initiates and potentiates the pathogenesis of COPD. Current research shows that HMGB1, RAGE and TLR4 are key pathways for the formation of CS‐induced COPD inflammation. Most studies have shown that HMGB1 plays a role in immune inflammation in smoking‐related COPD by directly binding to RAGE or TLR4, but HMGB1 may also activate proinflammatory cytokines through other pathways, such as the NFκB and JNK/p38 pathways via MyD88‐mediated pathways and lead to airway inflammation. The abnormal expression of RAGE and TLR4 also contributes to the accumulation of inflammatory cells and the release of inflammatory factors, which further activate the function of the HMGB1 and RAGE/TLR4 signaling pathways in the process of CS‐induced COPD inflammation. Besides, HMGB1, RAGE and TLR4 have potential to become clinical biomarker and treatment target of inflammation in COPD.

## CONFLICT OF INTEREST

The authors declare no conflict of interest.

## Data Availability

All publications discussed in the manuscript are available from the corresponding author on request.
